# Superior Functional Outcome and Comparable Health-Related Quality of Life after Enhanced Recovery vs. Conventional THA: A Retrospective Matched Pair Analysis

**DOI:** 10.3390/jcm10143096

**Published:** 2021-07-14

**Authors:** Franziska Leiss, Melanie Schindler, Julia Sabrina Götz, Günther Maderbacher, Matthias Meyer, Jan Reinhard, Florian Zeman, Joachim Grifka, Felix Greimel

**Affiliations:** 1 Department of Orthopedics, University Medical Center Regensburg, Asklepios Klinikum Bad Abbach, Kaiser-Karl-V.-Allee 3, 93077 Bad Abbach, Germany; f.leiss@asklepios.com (F.L.); m.schindler@asklepios.com (M.S.); ju.goetz@asklepios.com (J.S.G.); Guenther.maderbacher@ukr.de (G.M.); ma.meyer@asklepios.com (M.M.); j.reinhard@asklepios.com (J.R.); j.grifka@asklepios.com (J.G.); 2 Center for Clinical Studies, University Medical Center Regensburg, Franz-Josef-Strauss-Allee 11, 93053 Regensburg, Germany; florian.zeman@ukr.de

**Keywords:** total hip arthroplasty, THA ERAS, enhanced recovery, fast track, functional outcome, HRQoL

## Abstract

Background: The concept of enhanced recovery after total hip arthroplasty is gaining worldwide interest, as it shortens the length of hospital stay without an increase of complications. The aim of the study was to investigate the functional outcome and health-related quality of life 12 months after cementless total hip arthroplasty with the use of an enhanced recovery concept in comparison to a conventional rehabilitation. Material and Methods: 320 patients were retrospectively analyzed who underwent primary cementless total hip arthroplasty (THA). A total of 123 of the patients received an enhanced recovery program (ERAS) and 197 patients a conventional rehabilitation (Non-ERAS). Twelve months postoperatively, a clinical examination was performed regarding satisfaction, function and pain. Results were evaluated using WOMAC, EQ-5D-5L and EQ-VAS. A 1:1 matching was performed to correct for confounding variables, regarding age, sex and ASA score. Finally, 122 patients (*n* = 61, in each group) were analyzed and compared. Results: Patients showed a significant improvement of WOMAC total score, subscale pain, subscale stiffness and subscale function from preoperative to the follow up after 12 months in both groups, with significantly superior results for the WOMAC total score for the ERAS group (*p* = 0.042). EQ-5D and EQ-5D VAS showed a significant improvement from preoperative to 12 months postoperative (*p* < 0.001) for both groups, while no difference regarding the group-comparison was shown. Conclusion: Health-related quality of life and functional outcome increased to excellent values after total hip arthroplasty with the use of an enhanced recovery concept and a conventional rehabilitation, with a superior WOMAC total score for ERAS and a tendency to better results for health-related quality of life for patients with ERAS within the follow up after 12 months.

## 1. Introduction

The rising prevalence of osteoarthritis has led to an increase in primary total hip arthroplasty (THA) over the past two decades [1]. Since 2000, the number of hip replacements has increased rapidly between 2007 and 2017 by 30% [1]. In recent years, the concept of enhanced recovery (ERAS) has become established and is in intense discussion in the literature [2,3,4,5]. The concept was initially described by Kehlet [6]. It includes the optimization of logistical and organizational aspects, a structured management and implementation of a multimodal opioid sparing pain therapy. Advances in understanding of perioperative pathophysiology have led to an acceleration of the recovery process [7]. The introduction of ERAS in THA has led to a reduction of length of hospital stay, without an increase in complications, mortality and readmissions [2,4,8,9]. Furthermore, it could be shown that ERAS can also be used for elderly patients or patients with comorbidities [10,11]. Early mobilization of the patient on the day of surgery leads to an improvement of pulmonary function, less loss of muscle mass and function and reduces thromboembolic complications [12,13]. Information and education of patients about the operation procedure and perioperative care is a key issue of ERAS.

THA has shown to be an effective operation for symptomatic osteoarthritis. Despite its efficacy, 7–15% of patients still express dissatisfaction after the procedure [14]. The reasons for dissatisfaction after the procedure are diverse and can include persistent unexplained pain, unimproved walking ability or dissatisfaction due to unrealistic patient expectations [14,15]. Patient-reported outcomes (PROs) are an important tool for evaluating outcome and satisfaction after THA, as patients and surgeons may assess outcomes after THA differently.

The aim of the study was to evaluate the functional outcome and health-related quality of life after a primary cementless total hip arthroplasty with an enhanced recovery concept in comparison to a conventional rehabilitation. We hypothesized that patients with an enhanced recovery protocol would show a better functional outcome and higher postoperative satisfaction.

## 2. Materials & Methods

In this retrospective study, 320 patients were included who underwent primary cementless, collarless THA (DePuy Corail^®^ femoral stem, DePuy Pinnacle ^®^ acetabular component) between mid 2018 and mid 2019 in a single center. Inclusion criteria were primary THA using a DePuy Corail^®^ femoral stem due to primary or secondary osteoarthritis and a complete dataset of the collected scores. In the chosen period between 2018 and 2019, the ERAS setup was established parallelly in a part of the unit, and therefore, both setups could be ideally compared. Since both concepts were offered at the same time, the patient was able to choose one independently after knowing the differences between the treatment concepts. For all patients, an anterolateral approach was used. Exclusion criteria were revision surgery, severe congenital disorder of the hip, obesity III° (BMI > 40 kg/m^2^), malignancy and immobility. The data used were taken from digitized patients records. The local ethics committee granted ethical approval (reference number 19-1352-104). The study was applied in accordance with the ethical standards of the Declaration of Helsinki 1975.

The ERAS group consisted of 123 of the 320 THA-patients. The ERAS group received a patient education and preoperative gait training. One hour before surgery, a non-steroid-anti-inflammatory-drug (etoricoxib 90 mg) was administered. As an anesthetic technique, a short-acting spinal anesthesia was used (prilocaine 1% hyperbaric 4 mL with sufentanil 10 µg and dexamethasone 8 mg i.v. as the standard). Furthermore, tranexamic acid was administered topically (2 g) and intravenously (1 g). A local-infiltration analgesia was applied in the periacetabular and femoral region, as well as subcutaneously (200 mg ropivacaine for the deep periarticular infiltrations, with 0.5 mg adrenalin). In general, drains were not used. Postoperatively, full weight-bearing of the operated extremity was permitted. The first mobilization of the patient took place 2–3 h after surgery. Physiotherapeutic treatment was performed twice a day during the hospital stay and included exercises for muscle strengthening, tutorials to improve coordination and gait training under hip precautions. The physiotherapists were specially educated for the concept of ERAS. A treatment protocol for THA with the concept of ERAS was established in our department.

A total of 197 of 320 patients received a conventional recovery program after THA (non-ERAS group). As an anesthetic technique, a long-acting spinal anesthesia was used (bupivacain 0.5% = 4 mL). In contrast to the ERAS group, no NSAID was administered preoperatively. No tranexamic acid or local anesthesia was applicated during surgery. In general, wound drains were used. Patients were instructed to walk with crutches with full weight-bearing, according to the traditional postoperative recommendations of the unit. The first physiotherapeutic treatment was performed on the first postoperative day and took place once a day in consideration of hip precautions.

For optimizing postoperative pain, a pain management concept was established regarding the recommendations within the WHO analgesic ladder [16]. For the groups of ERAS and non-ERAS, the pain management was used equally. There was no shortening of the hospital stay, as we did not force this goal. The hospital stay was 5–7 days in all cases. General data on age, sex, BMI, operated leg, ASA-score (American Society of Anesthesiologists) and comorbidities were collected from the records. Comorbidities were represented by the Elixhauser Comorbidity score (ECM). The ECM originally counts 30 unweighted variables.

In our department, all THA patients were encouraged to complete several scores like the EQ-5D-5L, EQ-5D VAS and WOMAC. Euroqol-5D-5L has five dimensions of health status, with mobility, self-care, usual activities, pain/discomfort and anxiety/depression. Depending on the level, a digit is assigned to each dimension, so that you get a five-digit number combination (11111 = 1.000, representing full health and dead 00000 = 0). Euroqol VAS (EQ VAS) has a scale of 0–100 points. A score of 0 points represents the worst possible health status, while 100 points represents the best possible health status.

The Western Ontario and McMaster Universities Osteoarthritis Index (WOMAC) score includes 24 questions with respect to three subscales, i.e., pain, stiffness and physical function. The WOMAC Likert version was used, with a scale from 0 to 4 points for each question. The ranges of scores for the respective subscales are as follows: pain 0–20 points, stiffness 0–8 points and physical function 0–68 points, with the higher the value, the worse the result. All cases were reevaluated 12 months postoperatively, including a physical examination and evaluation of EQ-5D-5L, EQ-VAS and WOMAC.

## 3. Statistical Analysis

**Figure 1:**
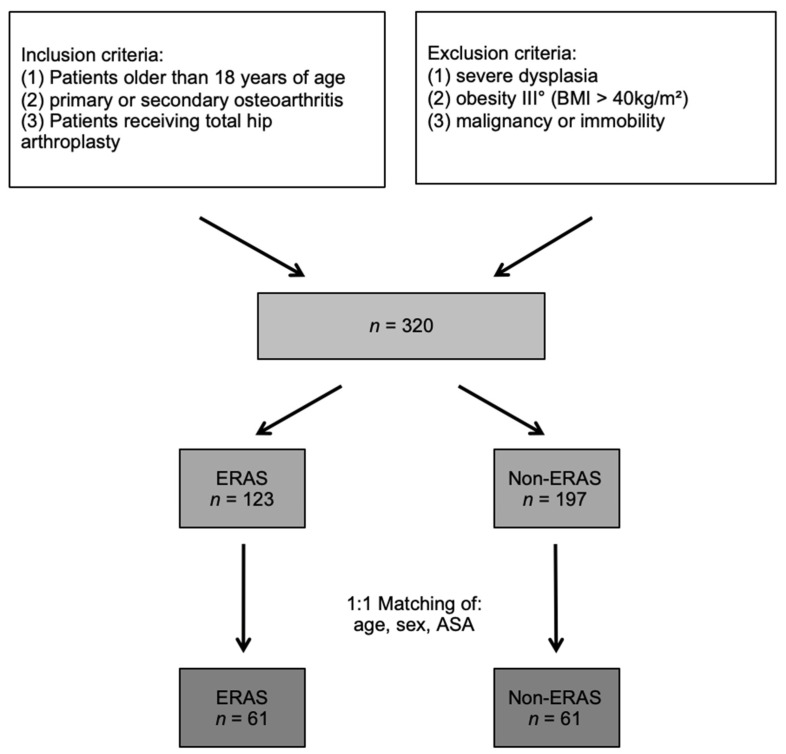
Flowchart: Enrollment and matched pair method of the study group.

The groups of ERAS and non-ERAS were adjusted in accordance with the matched pair method to get comparable groups in size and a distribution of confounders. The 1:1 matching was performed according to age, sex and ASA score. If there was more than one matching partner for one patient, one patient was randomly chosen. Thus, a total of 122 patients (*n* = 61, in each group, Figure 1) could be selected.

Normally distributed data were represented by mean values and standard deviation. The median (q1, q3) was used for non-normally distributed continuous or ordinal variables, while absolute and relative frequencies were used for categorial variables. A comparison of the scores from preoperative to postoperative were performed by using Wilcoxon signed rank test. Differences between the two groups regarding the quality of life measurements were assessed by using the Mann–Whitney-U-test. No adjustments of the significance level for multiple comparisons were done. The significance level was set to *p* < 0.05 for all tests. The statistical data were evaluated in SPSS 26.0 (IBM SPSS Statistics, Armonk, NY, USA—IBM Corp.).

## 4. Results

**Figure 2:**
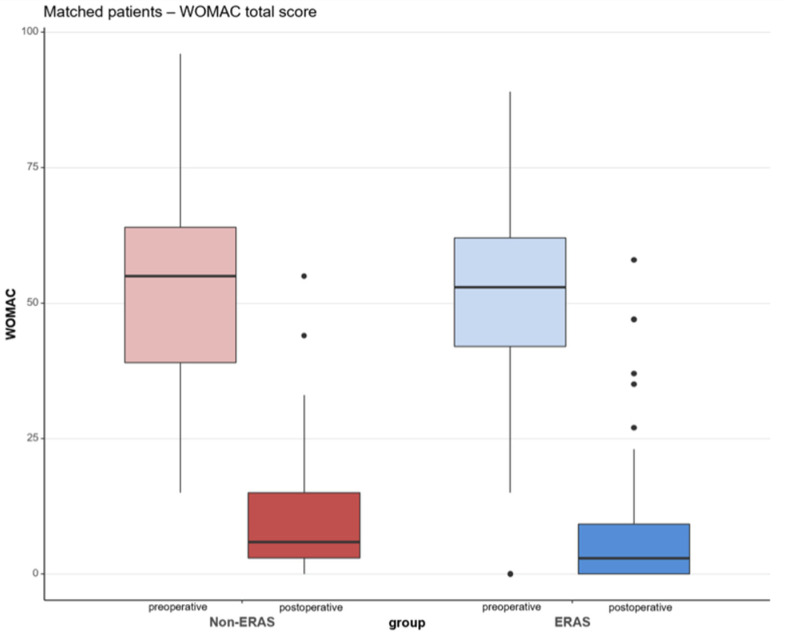
WOMAC Score (Western Ontario and McMaster Universities Osteoarthritis index) at the time preoperatively (pre) to 12 months postoperatively (post). Shown in red color: non-ERAS group; in blue color: ERAS group.

After adjusting the ERAS group and the non-ERAS group in accordance with the matched pair method for age, sex and ASA-score, a total of 122 patient (*n* = 61 per group, respectively) could finally be statistically analyzed (Figure 1). The specifics before and after matching of both the groups are shown in Table 1 and Table 2. The median WOMAC total score was 55.00 (IQR: 39.00, 64.00) preoperatively and improved until the follow up after one year to 6.00 (IQR: 3.00, 15.00) for the non-ERAS group. Regarding the ERAS group, the median WOMAC total score improved from 53.00 (IQR: 42.00, 62.00) preoperatively to 3.00 (IQR: 0.00, 9.25) after 12 months. There was a significant difference in the postoperative total WOMAC score between the ERAS and non-ERAS group, with *p* = 0.042 (Table 3), with the ERAS group showing a superior score compared to the non-ERAS group. There was no statistically significant difference between the two groups of ERAS and non-ERAS regarding the subscales of pain, stiffness and physical function preoperatively and postoperatively (Table 3). However, the ERAS group tended to show better values in all subscales. The subscales of pain, stiffness and physical function showed a significant improvement from preoperative to 12 months postoperative for both groups (*p* < 0.001) (Figure 2).

**Figure 3:**
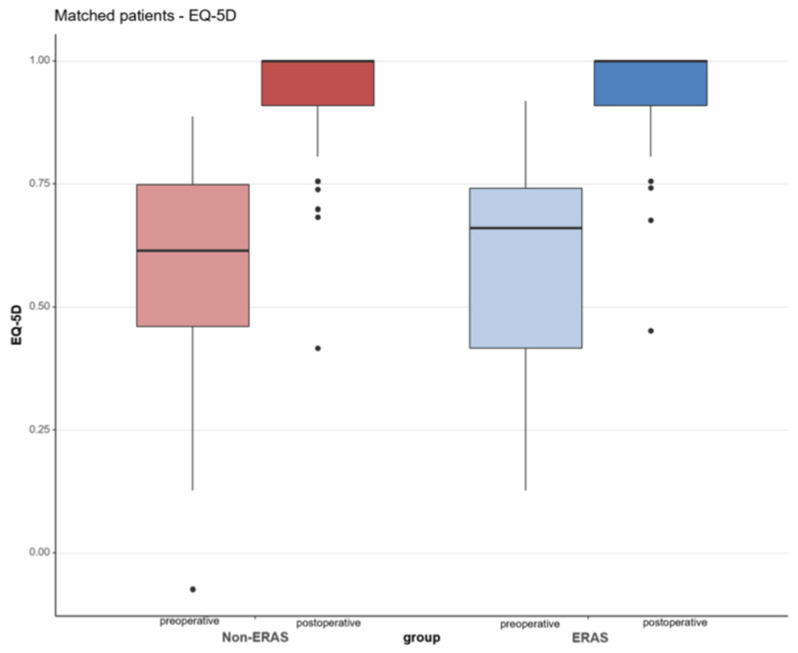
Euroqol (EQ-5D) with its five dimensions of mobility, self-care, usual activities, pain/discomfort and anxiety depression, at the time preoperatively (pre) and 12 months postoperatively (post). Shown in the red color: non-ERAS group; blue color: ERAS group.

The median Euroqol 5D-5L (EQ-5D) preoperatively showed no significant difference between the two groups (*p* = 0.889). The median EQ-5D preoperatively was 0.62 (IQR 0.46; 0.75) for the non-ERAS group and 0.66 (IQR 0.42; 0.74) for the ERAS group. Twelve months postoperatively, the ERAS and the Non-ERAS group improved for EQ-5D to 1.00 (0.91; 1.00), respectively, Table 3. The improvement from preoperative to postoperative was statistically significant for both groups (*p* < 0.001, respectively), Figure 3.

The median EQ-5D VAS preoperatively was 40.00 (IQR 30.00; 70.00) for the non-ERAS group and for the ERAS group 50.00 (IQR 36.25; 70.0). The EQ-5D VAS preoperatively was statistically not significant between the two groups (*p* = 0.183). The non-ERAS group improved 12 months postoperatively for EQ-5D VAS to 85.00 (IQR 75.00; 95.00), and the ERAS group improved to 90.0 (IQR 80.00; 95.00). An overview of the values of EQ-5D VAS is shown in Table 3. The improvement for EQ-5D VAS from preoperative to postoperative was statistically significant for both groups (*p* < 0.001, respectively).

The subgroups of EQ-5D with mobility, self-care, usual activities, pain/discomfort and anxiety/depression showed no significant difference regarding the comparison of ERAS and non-ERAS from preoperatively to postoperatively.

## 5. Discussion

A very high patient satisfaction could be shown both for the ERAS group and the non-ERAS group after total hip arthroplasty, with a significantly superior WOMAC total score for the ERAS group. In both groups, an improvement of 44 points in the WOMAC total score was achieved from preoperative to 12 months postoperative. A tendency towards better values in subscales of pain, function and stiffness of the WOMAC score was shown in the ERAS group. In the study of Yeo et al. [17], a minimally clinically important difference (MCID) for the WOMAC Score of 10.8 was calculated, and they stated that the WOMAC score can be used to determine clinically meaningful improvement. Regarding a conventional rehabilitation after THA, our results were similar to Yeo et al. [17], with a mean improvement in the WOMAC total score of 37 points from preoperative to postoperative after a follow up of 2 years of 1334 patients [17]. No studies on THA with an ERAS concept using the WOMAC score for assessing outcome could be found in the literature. We think that at an earlier follow-up time point after, for example, 4–6 weeks or 3 months would show a greater difference between the two groups, because after a follow up of one year, the results of the post-treatment have probably adapted. A shortening of length of hospital stay without compromising quality and results of the treatment can be achieved by optimizing the interdisciplinary process [2]. The focus of this study was to consider functional outcome and health-related quality of life after THA, rather than a possible shortening of the length of hospital stay. Bellamy et al. [18] presented population-based normative values of the WOMAC score for the subscales of pain, stiffness and function. In the age groups of 50–74, the subscale of pain had a value between 1.36–1.79, the subscale of stiffness had a value between 2.03–2.44 and the subscale of function had a value between 1.28–2.07. The average scores for pain, stiffness and function were 1.41 (SD ± 1.97), 2.01 (SD ± 2.36) and 1.54 (SD ± 2.02), respectively. Comparing the population-based normative values of the WOMAC score to our study collective, there were higher values in all subscales preoperatively for our study collective. In the 12 months follow-up, the value of the subscale pain showed a higher value of 1.59 for the group of non-ERAS than the average score of the normative value, but for the ERAS-group, a value with 1.34 was shown, being inferior to the normative value. For the subscale of stiffness, both groups postoperatively showed values inferior to the normative value. Regarding the subscale function, both groups still had superior values to the comparative norm in the follow up after 12 months, with a tendency towards inferior values for the group of ERAS. This raises the question whether an adaptation of physiotherapeutic aftercare is generally necessary, e.g., in addition to an enhanced recovery concept. Comorbidities of the study collective, such as further osteoarthritis of other joints, which could limit the function, also might play a role.

The health-related quality of life (HRQoL) was measured by Euroqol (EQ-5D). From preoperative to the follow up after 12 months, the improvement of EQ-5D was statistically significant (*p* < 0.001) for both groups, ERAS and Non-ERAS. In the follow up after 12 months, both groups had a median EQ-5D of 1.0 for the ERAS and non-ERAS groups. The studies of Larsen et al. [19] and Aalund et al. [20] showed comparable results to our study, with a continued rise of the values of EQ-5D to the follow up after 12 months. Preoperatively, both groups showed an inferior score compared to the population norm of Germany. The EQ-5D population index norm for Germany is 0.915 for the age of 55–64 and 0.882 for the age of 65–74, and in total for all age groups, it is 0.93 [21]. Postoperatively, patients of both groups even reached a superior value in relation to the population norm. The Swedish Hip Arthroplasty Register (SHAR) is a register that monitors HRQoL by using the EQ-5D as a standard [22]. Our results for mean HRQoL of 0.94 for the ERAS group and 0.93 for the non-ERAS group one year postoperatively are higher than their reported average national value of 0.76 and higher than the hospital, with the highest average score of 0.86 [22]. Larsen et al. [19] showed, in their study of 196 patients with ERAS, a mean HRQoL of 0.90; in their opinion, the difference was mainly caused by the fast-track intervention. In our study, both the ERAS group and the non-ERAS group showed values above the population norm, which is probably attributable to patient selection and the small study collective. The deviation is not explained by a selection of patients with few secondary diseases (compare Elixhauser Comorbidity score, Table 2).

The EQ-VAS ratings in both groups showed a significant improvement from preoperative to postoperative, with a mean of about 35 points. The EQ-VAS population norm rates a value of 77.3 for the total population in Germany; for the age group of 55–64, it rates a value of 72.9 and for the age group of 65–74, it rates a value of 68.8. Similar to EQ-5D, the EQ-VAS showed better results for both groups than the population norm [21].

Considering the costs and risks of the procedure, the goal should be at least to reach the population norm values at the earliest possible stage. Furthermore, this should be achieved with as little risk of complications and pain as possible. We think that the concept of enhanced recovery has the possibility to achieve this goal at an early stage.

The present study has some limitations. The first is the retrospective and record-based design. To minimize confounders between the groups, a matching was performed. Second, the study collective was relatively small, young (average age: 63 years) and relatively fit (predominantly ASA was 2). Furthermore, the ERAS concept was newly established in our department, which could have caused a selection bias. As the maximum follow up in this study was limited to 12 months, long-term results are not available. Another point which could have influenced the results is the difference of inpatient rehabilitation or outpatient rehabilitation. There was no direct comparison between the two rehabilitations. A randomized controlled study design or further prospective long-term studies should be undertaken to measure and quantify health-related quality of life and functional outcome after total hip arthroplasty with the concept of enhanced recovery.

## 6. Conclusions

Total hip arthroplasty with the use of an enhanced recovery concept and a conventional rehabilitation showed a high patient satisfaction in the present study. In a follow-up period of 12 months, pain scores decreased significantly, and HRQoL scores increased to excellent values, and even higher values than the population norm were achieved. We could show that an ERAS concept tends to lead to a better outcome after THA compared to a conventional concept, with statistically significant superior functional scores (WOMAC total score) in the matched study population.

## Figures and Tables

**Table 1:** Demographic and general data before matching.

	Eras	Non-Eras	All
Patients, total (*n*)	123	197	320 (100%)
Age in years (mean ± SD)	60.7 ± 10.32	67.7 ± 9.41	64.5, 65.0 ± 10.32
Sex in % (female:male)	35.0:65.0	58.9:41.1	49.7:50.3
ASA score	1: 27.6%	1: 16.7%	1: 20.6%
	2: 66.7%	2: 62.9%	2: 64.4%
	3: 5.7%	3: 20.8	3: 15.0%
	4: 0	4: 0	4: 0%
Side of operation	Right: 52.8%	Right: 52.8%	Right: 52.5%
	Left: 48.0%	Left: 47.2%	Left: 47.5%
Body Mass index (kg/m^2^) (mean ± SD)	28.2 ± 4.33	29.2 ± 5.24	28.8 ± 4.93
ECM	0: 36.6%	0: 17.8%	0: 25.0%
	1: 36.6%	1: 27.9%	1: 31.3%
	2: 19.5%	2: 32.5%	2: 27.5%
	>3: 7.3%	>3: 21.8%	>3: 16.3%

Abbreviations: ASA: American Society of Anesthesiologists, ECM: Elixhauser Comorbidity Method. Continuous variables are given as mean values/±standard deviation; categories are in absolute numbers/percent.

**Table 2:** Demographic and general data after matching.

	Eras	Non-Eras	All
Patients total (%)	61 (50%)	61 (50%)	122 (100%)
Age in years (mean ± SD)	63.1 ± 8.34	63.1 ± 8.34	63.1 ± 8.34
Sex in % (female:male)	50.8:49.2	50.8:49.2	50.8:49.2
ASA score	1: 18.0%	1: 18.0%	1: 18.0%
	2: 78.7%	2: 78.7%	2: 78.7%
	3: 3.3%	3: 3.3%	3: 3.3%
	4: 0%	4: 0%	4: 0%
Side of operation	Right: 52.5%	Right: 52.5%	Right: 52.5%
	Left: 47.5%	Left: 47.5%	Left: 47.5%
Body Mass index (kg/m^2^)	27.9 ± 4.65	30.1 ± 4.67	29.0 ± 4.78
ECM	0: 39.3%	0: 24.6%	0: 32.0%
	1: 31.1%	1: 39.3%	1: 35.2%
	2: 24.6%	2: 27.9%	2: 26.2%
	>3: 4.9%	>3: 8.2%	>3: 6.6%

Abbreviations: ASA: American Society of Anesthesiologists, ECM: Elixhauser Comorbidity Method. Continuous variables are given in mean values/±standard deviation; categories are in absolute numbers/percent.

**Table 3:** PROM scores of Euroqol (EQ-5D), Euroqol VAS (EQ VAS) and WOMAC (Western Ontario and McMaster Universities Osteoarthritis Index) preoperatively (pre.) and 12 months (follow up) after surgery. Median values and interquartile range (IQR).

	Median (IQR)		*p*-Value
	Non-Eras	Eras	
EQ-5D pre.	0.62 [0.46, 0.75]	0.66 [0.42, 0.74]	0.889
EQ-5D Follow up	1.00 [0.91;1.00]	1.00 [0.91;1.00]	0.753
EQ VAS pre	40.00 [30.00;70.00]	50.00 [36.25;70.0]	0.183
EQ VAS Follow up	85.00 [75.00;95.00]	90.0 [80.00;95.00]	0.753
WOMAC pain pre.	11.00 [9.00, 14.00]	12.00 [10.00, 13.50]	0.696
WOMAC pain Follow up	0.00 [0.00, 2.00]	0.00 [0.00, 2.00]	0.562
WOMAC Stiffness pre.	5.00 [4.00, 6.00]	5.00 [4.00, 6.00]	0.311
WOMAC Stiffness Follow up	1.00 [0.00, 2.00]	0.00 [0.00, 2.00]	0.529
WOMAC Function pre.	38.00 [28.00, 46.00]	37.00 [27.50, 42.00]	0.539
WOMAC Function Follow up	4.00 [2.00, 10.00]	3.00 [0.00, 9.00]	0.222
WOMAC total pre.	55.00 [39.00, 64.00]	53.00 [42.00, 62.00]	0.558
WOMAC total Follow up	6.00 [3.00, 15.00]	3.00 [0.00, 9.25]	0.042

## Data Availability

Data is available at the authors institution.
